# Gold Nanotapes and Nanopinecones in a Quantitative Lateral Flow Assay for the Cancer Biomarker Carcinoembryonic Antigen

**DOI:** 10.1021/acsanm.3c03053

**Published:** 2023-09-19

**Authors:** Joseph Fox, Damien V. B. Batchelor, Holly Roberts, Samuel C.T. Moorcroft, Elizabeth M.A. Valleley, Patricia Louise Coletta, Stephen D. Evans

**Affiliations:** † Molecular and Nanoscale Physics Group, School of Physics and Astronomy, University of Leeds, Leeds LS2 9JT, United Kingdom; ‡ Leeds Institute of Medical Research, Wellcome Trust Brenner Building, St James’s University Hospital, Leeds LS9 7TF, United Kingdom

**Keywords:** gold, nanoparticles, lateral flow assay, cancer, biomarker, hierarchical nanoparticles, quantitative, carcinoembryonic antigen

## Abstract

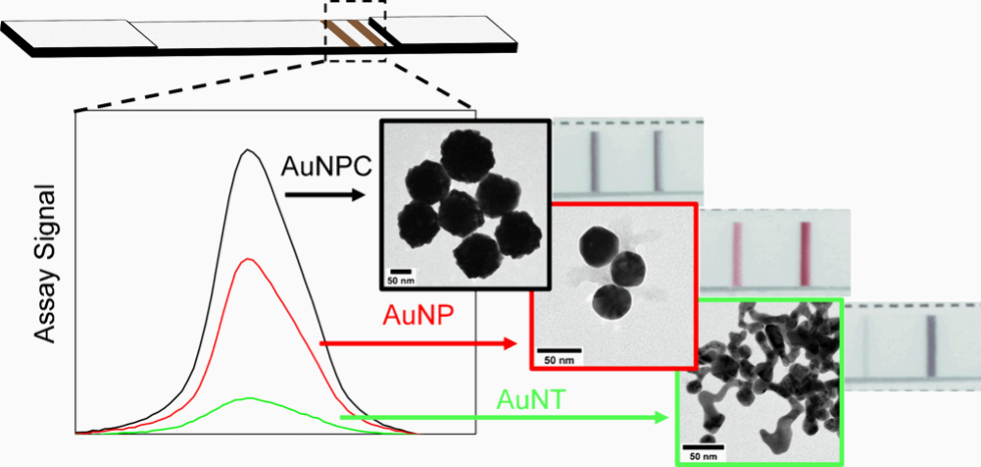

Colorectal cancer is the third most common malignancy and the second leading cause of cancer death globally. Multiple studies have linked levels of carcinoembryonic antigen in patient serum to poor disease prognosis. Hence, the ability to detect low levels of carcinoembryonic antigen has applications in earlier disease diagnosis, assessment, and recurrence monitoring. Existing carcinoembryonic antigen detection methods often require multiple reagents, trained operators, or complex procedures. A method alleviating these issues is the lateral flow assay, a paper-based platform that allows the detection and quantification of target analytes in complex mixtures. The tests are rapid, are point-of-care, possess a long shelf life, and can be stored at ambient conditions, making them ideal for use in a range of settings. Although lateral flow assays typically use spherical gold nanoparticles to generate the classic red signal, recent literature has shown that alternate morphologies to spheres can improve the limit of detection. In this work, we report the application of alternative gold nanoparticle morphologies, gold nanotapes (∼35 nm in length) and gold nanopinecones (∼90 nm in diameter), in a lateral flow assay for carcinoembryonic antigen. In a comparative assay, gold nanopinecones exhibited a ∼2× improvement in the limit of detection compared to commercially available spherical gold nanoparticles for the same antibody loading and total gold content, whereas the number of gold nanopinecones in each test was ∼3.2× less. In the fully optimized test, a limit of detection of 14.4 pg/mL was obtained using the gold nanopinecones, representing a 24-fold improvement over the previously reported gold-nanoparticle-based carcinoembryonic antigen lateral flow assay.

## 1 Introduction

Colorectal cancer (CRC) is the third most common malignancy and the second leading cause of cancer death globally,^1^ with 1.8 million new cases diagnosed in 2018.^2^ First discovered by Gold and Freedman^3^ in 1965, carcinoembryonic antigen (CEA) is an antigen produced by many human tumors, including breast, lung, and colorectal cancer.^4^ Multiple studies have linked raised CEA levels to poor disease prognosis.^5−9^ As CEA is upregulated in ∼90% of advanced CRC,^10^ routine CEA blood tests are taken after curative-intent surgery, with 3–7 ng/mL^11^ usually prompting further investigation. Hence, the ability to detect CEA (and other cancer biomarkers) permits earlier disease diagnosis, assessment, and recurrence monitoring^12^ while opening avenues for novel treatment timing strategies.^13^

CEA is a membrane protein, part of which is cleaved and secreted into the blood and, as such, can be detected by noninvasive liquid biopsy (blood sample).^14^ This is preferred over tissue biopsy, particularly for CRC, in which tissue extraction may be impossible due to clinical complications and/or cost.^15^ The release of CEA into the blood creates the opportunity for detection by enzyme-linked immunosorbent assay (ELISA), with commercially available systems providing a limit of detection (LOD) of 0.2 ng/mL.^16^ In a clinical setting, CEA in serum can be quantified using automated immunoassay systems such as the Advia Centaur XP (chemiluminescence) and Elecsys E170 (electrochemiluminescence) with LODs of 0.5 and 0.2 ng/mL, respectively.^17^ A range of novel techniques have been applied, including the use of dsDNA templated copper nanoparticles,^18^ a label-free electrochemiluminescence aptasensor,^19^ and a gold nanoparticle (AuNP) chemiluminescence system^20^ to provide LODs of 0.0065, 0.0038, and 0.034 ng/mL CEA in serum, respectively.

Although these methods obtain high sensitivity, they often require trained lab operators and/or lengthy/complex procedures. A method alleviating these issues and that is the focus of this study is the lateral flow assay (LFA). LFAs are a paper-based platform for the detection and quantification of analytes in complex mixtures.^21^ LFAs are point-of-care, are low cost, have a long shelf life (without refrigeration), and, as such, are ideal for use in developing countries and remote regions.^21^ Spherical AuNPs are widely used as LFA labels, generating the visible signal in many commercially available LFA kits.^22^ The accumulation of AuNPs at the test line (TL) and control line (CL) generates a deep red color that can be used for naked-eye qualitative tests,^23^ or signal intensity can be analyzed for quantitative analysis.^24^ Zeng et al.^25^ used 20 nm AuNPs in an LFA, which obtained a 5 ng/mL LOD for CEA in buffer and human plasma. Mahmoudi et al.^26^ utilized 11 nm AuNPs and “oriented” CEA antibodies at the test line to achieve an LOD of 0.35 ng/mL for serum samples. Studies have also been conducted using nongold nanoparticle labels, with magnetic-nanoparticle-based lateral flow assays achieving LODs for CEA in serum of 0.27^27^ and 0.40 ng/mL.^28^

The recent literature has shown that alternate morphologies to spherical AuNPs can improve the LOD. For example, Zhang et al.^29^ compared “flowerlike” (280 nm or 3 μm), “popcornlike” (180 nm), and spherical (20 or 40 nm) AuNPs for *E. coli* detection. The small “flowerlike” probes (280 nm) displayed the greatest sensitivity, allowing detection down to 10^3^ CFU/mL. Serebrennikova et al.^30^ tested gold “nanostars” and “nanopopcorns” for the detection of a bacterial infection marker, with “nanopopcorns” providing a 0.1 ng/mL detection limit (5× improvement over 20 nm AuNPs).^30^ Lai et al.^31^ compared 30 nm spherical AuNPs to gold “nanoflowers” (20–35 nm) for the detection of a prohibited meat leanness enhancer in swine urine. The “nanoflowers” gave a 12.5 pg/mL LOD, ∼5× lower than spherical AuNPs.^31^ These enhancements are attributed to rough nanoparticles having more favorable surfaces for antibody adsorption and the high surface area to volume ratio afforded by hierarchical structures.^30,31^

We previously reported the synthesis^32^ and optimization^33^ of free-standing, subnanometer (two atomic layers, 0.47 nm thick) 2D gold nanosheets (AuNS). In the current work, by variation to the one-pot, seedless AuNS synthesis, we produce additional novel nanoparticle morphologies, gold “nanotapes” (AuNTs) and gold “nanopinecones” (AuNPCs), both of which possess potentially desirable characteristics for LFA. AuNPCs (90 nm in diameter) display a hierarchical surface topology composed of clusters of the subnanometer 2D AuNS material, whereas AuNTs (∼35 nm in length) have a quasi-1D structure with a 3D “head” and 2D “tape” region. Although the above literature^29−31^ indicates that the rough AuNPC morphology would be desirable for LFA labels, the low dimensionality of AuNSs and AuNTs is also of interest.^34^

The signal of LFAs has been enhanced by using AuNP bound to the TL to catalyze the reaction between H_2_O_2_ and 3,3′,5,5′-tetramethylbenzidine (TMB)^35^ or 3,3′-diaminobenzidine (DAB)^36^ to yield colored products at the test line. Low dimensional nanoparticles were recently shown to display improved catalytic activity over higher dimensionality counterparts;^34^ this could be desirable for substrate amplification in LFAs following particle binding at the TL or CL.^35^

Further, methods of producing many of the different gold particle morphologies described in the literature require multistep seeded synthesis procedures for the generation of hierarchical^29−31^ and wire-like^37^ structures. In contrast, our AuNPCs and AuNTs are produced using a facile, one-pot synthesis route, which is conducted under ambient conditions and in aqueous solvents.

We have compared AuNPCs and AuNTs to spherical AuNP (40 nm) in an LFA for CEA detection. These nanoparticles are collectively termed AuNX throughout this work. Our assay uses commercially available BioPorto strips.^38^ BioPorto strips contain prefunctionalized TL and CL. Samples are mixed with a matched antibody pair for the target analyte (one biotinylated and one conjugated to AuNX), creating a complex that only forms in the presence of the target analyte. Upon application to the test strip, these complexes bind at the TL due to the interaction between biotin binding proteins immobilized at the TL and the biotinylated antibody in the antigen–antibody complex. The CL binds any antimouse/rabbit/goat antibody, confirming the sample has flowed correctly up the strip. The quantitative nature of the assay was assessed by determining the intensity of the color change due to particle binding at the test line.

## 2 Experimental Section

### 2.1 Materials

Gold(III) chloride trihydrate (520918), bovine serum albumin (BSA, A7638), rabbit IgG polyclonal antibody (PP64-pAb), TWEEN20 (P7949), H_2_O_2_ (30% w/w, 16911), potassium carbonate (791776), and 40 nm diameter AuNPs (741981) were purchased from Sigma. Methyl orange (MO, 17874) and trisodium citrate (45556) were purchased from Alfa Aesar. Hydrochloric acid (32%, H/1100/PB17), nitric acid (70%, N/2250/PB17), PBS (17–516F), and 20 mL borosilicate clear glass vials (14-955-313) were purchased from Fisher scientific. Ninety-six-well plates (655-180) were purchased from Greiner Bio-One. Generic LFA strips (gRAD OneDetection) and BioPorto buffer (SDB50) were purchased from BioPorto Diagnostics. Purified biotinylated mouse CEACAM5 monoclonal detection antibody (clone 1C2, TA700492), purified CEACAM5 mouse monoclonal capture antibody (clone 2B12, TA600492), and CEACAM5 human recombinant protein (TP710040) were purchased from OriGene Technologies. DAB substrate (1855900) and stable peroxide substrate buffer (1855910) were purchased from Thermo Scientific. Innova SARS-CoV-2 rapid antigen lateral flow qualitative test kit extraction solution (Innova buffer) was obtained through the NHS England self-testing kit. Milli-Q water (18.2 MΩ·cm at 25 °C) was used in all solutions/syntheses.

### 2.2 Synthesis of AuNX

All glass ware was cleaned using aqua regia prior to use, and reactions were conducted at room temperature. AuNTs were prepared by mixing 4 mL of 0.21 mM MO with 1 mL of 5 mM HAuCl_4_ followed by the addition of 0.5 mL of 100 mM sodium citrate (after 30 s). The mixture was left undisturbed for 17 h, then washed three times by centrifugation (3000*g* for 60 min), and finally resuspended in 1 mL of Milli-Q. AuNPCs were prepared by mixing 40 mL of 2.5 mM MO with 10 mL of 5 mM HAuCl_4_ followed by the addition of 5 mL of 150 mM sodium citrate (after 30 s). The mixture was left undisturbed for 17 h at room temperature. To clean, the synthesized product was split into 5 mL aliquots, and each aliquot was diluted with 5 mL Milli-Q, centrifuged three times at 4600*g* for 10 min, and finally resuspended in 1 mL of Milli-Q.

### 2.3 Characterization of AuNX

Absorbance spectra were taken using an Agilent Technologies Cary 5000 ultraviolet–visible–near-infrared (UV–vis–NIR) spectrophotometer. Transmission electron microscopy (TEM) imaging was conducted using two systems: (i) a Tecnai G2 Spirit TEM (T12) operated at an acceleration voltage of 120 kV with a Lab6 filament and a Gatan Us4000 CCD camera for image capture and (ii) an FEI Tecnai TF20 FEGTEM operated at an acceleration voltage of 200 kV with a Gatan Orius SC600A CCD camera for image capture. The grids used for imaging were 400 mesh copper grids coated with an ∼8 nm-thick carbon support film (SPI Supplies). Samples were prepared by pretreating carbon-coated copper grids with PELCO easiGlow followed by adding 3 μL of nanoparticle solution with an optical density at 400 nm of 1 (OD_400_ = 1, Milli-Q) pipetted onto the grid and left to dry. Particles were sized using widefield TEM images and a custom ImageJ macro script that determines the area of each AuNPC and then approximates particles as circles to determine the radius. Scanning electron microscopy (SEM) imaging was conducted by using a Hitachi SU8230 at an operating voltage of 2 kV, with samples dried onto specimen stubs prior to imaging. To determine the concentration of Au in samples, atomic absorption spectroscopy (AAS) measurements were taken using an Agilent 240 fs atomic absorbance spectrophotometer. An air/acetylene gas mixture was used, and a lamp detected Au at 242.8 nm.

### 2.4 Preparation of Anti-CEA Conjugated AuNX

AuNX were conjugated to the anti-CEA monoclonal antibody (CEA-mAb) using slight variations to methods found in the literature.^35,39^ Briefly, 2.5 mL of AuNX (OD_400_ = 0.5, Agilent), 20 μL of 0.1 M K_2_CO_3_, and 20 μg of CEA-mAb were mixed, placed on a PMR-30 shaker for 30 min, and incubated at 4 °C overnight. After incubation, 250 μL 10% BSA solution in Milli-Q was added, and the solution was placed on a PMR-30 shaker for 1 h and then cleaned twice by centrifugation. AuNPC and AuNP conjugates were centrifuged at 4600*g* for 10 min, whereas AuNT conjugates were centrifuged at 3000*g* for 60 min. In all cases, after the first cleaning, pellets were resuspended in 2.5 mL of MQ, whereas after the final cleaning, the pellets were resuspended in 0.2 mL of pH adjusted Milli-Q (10 mL of Milli-Q + 80 μL of 0.1 M K_2_CO_3_). Samples were kept protected from light at each stage.

### 2.5 Comparing AuNX Morphologies in CEA LFA Tests

The 40 nm spherical AuNPs, AuNTs, and AuNPCs were conjugated to anti-CEA monoclonal antibodies following the procedure described above. LFAs were undertaken in a 96-well plate by combining 5 μL of bio-mAb at 40 μg/mL and 10 μL of AuNX-CEA-mAb conjugates (OD_400_ = 3.4, Agilent), with 100 μL of CEA solution at a range of concentrations in Innova running buffer. The mixture was incubated at room temperature for 5 min, and then test strips were dipped into the well for 15 min to allow the buffer to run up the strip prior to imaging. Signals were quantified using a custom MATLAB script, and three repeats of each condition were used, in which new dilutions of bio-mAb and CEA were made. LOD and limit of quantification (LOQ) were calculated using eqs 1 and 2,^40^ in which σ represents the standard deviation of the response (the residual standard deviation of a linear regression fit to the calibration curve) and *S* is the gradient of the calibration curve.
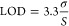
1
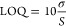
2

### 2.6 Enhancing LFA Sensitivity Using the DAB Substrate

AuNPCs were conjugated to anti-CEA monoclonal antibodies, as described above. LFAs were undertaken in a 96-well plate by combining 5 μL bio-mAb at 100 μg/mL and 20 μL AuNPC-CEA-mAb conjugates (OD_400_ = 2.5, Agilent) with 75 μL CEA solution at a range of concentrations in the BioPorto buffer. The test strip was dipped into the well for 10 min before moving the strip into a well containing 50 μL of BioPorto buffer and imaged after a further 5 min. Concentrations given refer to the final CEA concentration in the well. For enzymatic amplification, a reaction solution was prepared by mixing the DAB substrate, stable peroxide substrate buffer, and H_2_O_2_ at the volume ratio 1:1:2. Fifty microliters of the reaction solution was added to the TL and allowed to dry for 10 min before another 50 μL of the reaction solution was added and allowed to dry for a further 10 min. Signals were quantified using a custom MATLAB script, and three repeats of each condition were used.

## 3 Results and Discussion

### 3.1 Synthesis and Characterization of AuNX

**Figure 1:**
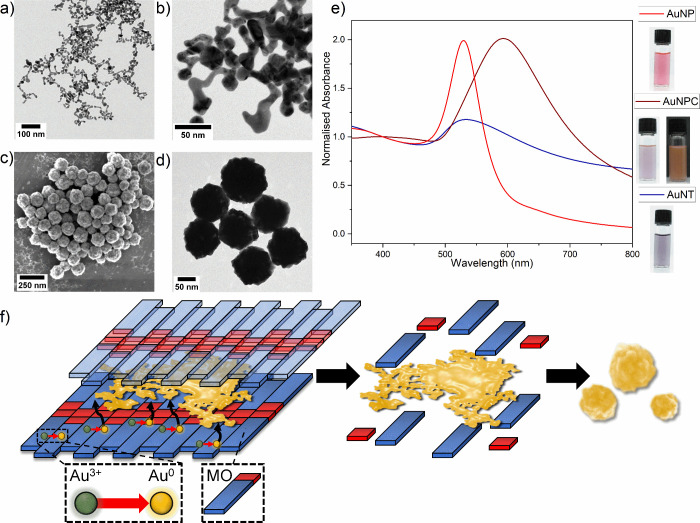
Synthesis and characterization of AuNX. (a) Widefield and (b) close-up TEM images of AuNTs. (c) SEM and (d) TEM of AuNPCs. (e) UV–vis spectra of AuNX normalized to 1 at 400 nm, with inset photographs of aqueous nanoparticle solutions at OD_400_ = 0.5. (f) Schematic showing suggested formation of 2D flakes and subsequently AuNPCs. Initially, 2D flakes form because of a templating effect when gold ions are reduced in the presence of planar stacks of the amphiphile MO. Second, MO is rapidly degraded, generating free 2D flakes. Finally, subsequent controlled aggregation and hierarchical assembly of 2D flakes result in the AuNPC structure.

Our previous work^33^ optimized the synthesis of 2D AuNS. It was shown that when using the same reagent concentrations for AuNS synthesis, stronger centrifugation speeds yielded tape-like nanoparticles, displaying a dense 3D head, as shown by TEM images (Figure 1a,b). Manual sizing of AuNTs gave an average tape length of 35.2 ± 16 nm (*N* = 430). UV–vis spectra of the AuNT (Figure 1e) show a broad plasmon peak centered at 533 nm, with a lower absorbance than seen for 3D AuNP but with greater absorbance extending into the NIR region. AuNT spectra show a maximum at 533 nm with OD_533_ = 0.727 (10× diluted) and OD_400_ = 0.617 (10× diluted). The presence of the distinct SPR band is attributed to the 3D “head” regions of the tapes. AAS of AuNT determined the total gold yield to be ∼48% with gold loss occurring during centrifugation.

We recently reported that by increasing the MO concentration used in AuNS synthesis, AuNPCs can be obtained.^34^ SEM (Figure 1c) and TEM (Figure 1d) of AuNPCs show size monodisperse particles with rough hierarchical structure. The ImageJ analysis of widefield TEM images of AuNPCs (Figure S1) gives a mean diameter of 89.6 ± 21 nm (*N* = 303). AuNPCs exhibit a broad UV–vis peak (Figure 1e) with a maximum at 593 nm, with OD_593_= 0.133 (100× diluted), OD_400_ = 0.0661 (100× diluted), and full width at half-maximum (fwhm) of 211 nm. AuNPCs were analyzed by AAS, and the total gold yield after cleaning was determined to be ∼70%. AuNPC synthesis was shown to be scalable and could be completed in 30 min with minimal impact on size, morphology, or spectral characteristics. AuNPs of 40 nm diameter were used for comparison in this study, and as such, a representative UV–vis spectrum has been included in Figure 1e. UV–vis spectra for AuNT and AuNPC were unchanged throughout the course of the LFA experiments, indicating good colloidal stability over the experimental time scales. Further studies showed stability when stored at 20 °C for >5 and >8 months for AuNTs and AuNPCs, respectively, as shown in Figure S2. After long-term storage, an unexpected shift in AuNPC spectra to shorter wavelengths is observed.

The AuNPC solutions (Figure 1e, inset) exhibit dichroic properties. When illuminated uniformly, the AuNPCs appear blue-purple; however, when illumination from the rear is blocked, the AuNPC solution shows a pink-brown coloration. AuNTs gave a blue-purple aqueous solution (Figure 1e, inset). The generation of a range of alternate colors to the red line typically observed for colloidal gold is potentially desirable for LFA. Nonred colors give the potential for enhancing the signal-to-noise ratio when using a blood sample while also being useful for an array of multicolored test lines in a multiplexed assay.^41^ For synthesis of AuNSs, AuNTs, and AuNPCs, MO is used as a confining agent to dictate the final morphology of the reduced gold, as discussed in our previous work.^32−34^ It has previously been suggested that AuNPCs form when small flakes of 2D AuNS material, formed by a templating effect caused by self-assembly of the amphiphile MO,^32,33^ subsequently undergo controlled aggregation and hierarchical assembly to arrange in stacked layers,^34^ as outlined in Figure 1f. AuNTs are potentially smaller, intermediate structures formed during 2D AuNS synthesis, which are readily collected at stronger centrifugation speeds.^33^

### 3.2 Optimization of LFA Parameters

**Figure 2:**
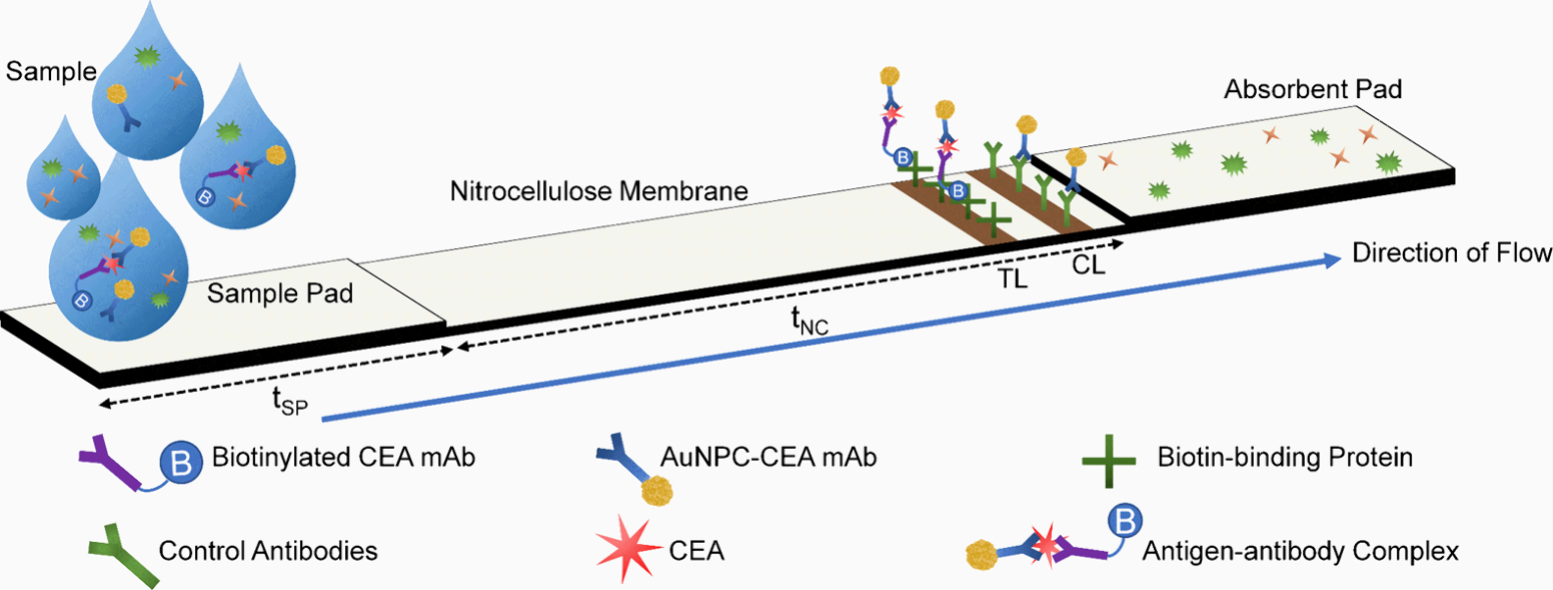
Schematic of the LFA test strip used in the study. As the sample flows up the test strip, complexes formed between the biotinylated capture antibody, CEA, and gold conjugated capture antibody collect at the test line, whereas all antibodies collect at the control line. The time taken for the liquid to travel through the sample pad and nitrocellulose section is defined by *t*_SP_ and *t*_NC_.

The working principle of our CEA LFA is presented in Figure 2. BioPorto strips are commercially available and are preloaded with a biotin-binding protein at the TL and a mixture of mouse, rabbit, and goat antibodies (nonspecific, control antibodies^38^) immobilized at the CL. The assay uses a wet conjugate method, in which the sample is mixed with a matched pair of anti-CEA monoclonal antibodies, one of which is biotinylated (biotinylated-CEA-mAb, shortened to bio-mAb throughout this work) and one of which is conjugated to our AuNX (AuNX-CEA-mAb). Biotinylated-CEA-mAb, AuNX-CEA-mAb, and CEA combine to create a complex that can form only in the presence of CEA.

The sample is then applied to the sample pad, and these complexes bind at the TL because of the interaction between the biotin binding protein immobilized at the TL and the biotinylated-CEA-mAb in the antigen–antibody complex. The nonspecific CL binds any antimouse/rabbit/goat antibody, confirming that the sample has flowed correctly up the strip. The accumulation of AuNX at the TL and CL provides a visible color change that was analyzed using a custom MATLAB script, outlined in Figure S3. All AuNX tested were shown to readily conjugate to anti-CEA antibodies, with minimal aggregation or alteration to the original AuNX spectra, except for slight red shifts, indicative of successful antibody conjugation,^35^ as shown in Figure S4.

A range of optimization experiments were conducted before the final assay parameters were selected. Parameter optimization showed equivalent findings across the different morphologies tested. Figure S5a shows images of the TL and CL signals for LFAs using different AuNP-antibody conjugate concentrations, run with blank BioPorto buffer. Peak prominence analysis (Figure S5b) shows that CL signal rises with increasing AuNP-antibody conjugate concentration until showing slight saturation at OD_400_ ∼ 2.5 (Agilent). In the final assay, we used the highest OD_400_ obtainable across all AuNX-antibody conjugates post conjugation and cleaning.

The bio-mAb enables binding to the TL in this assay. The bio-mAb concentration was optimized in Figure S6, in which we observe that the average TL peak prominence rises with bio-mAb concentration before slightly saturating after 40 μg/mL, with low nonspecific binding at this concentration. Hence, 40 μg/mL bio-mAb was selected.

It was shown that the inclusion or exclusion of BSA (Figure S7a) had a minor impact on TL and CL signals in running buffers tested, except for the BioPorto buffer. Figure S7a also shows that the choice of running buffer contributes significantly to LFA signal and nonspecific binding. This is attributed to the different buffers traveling through the test at different rates. The time taken for the liquid front to pass through the nitrocellulose (*t*_NC_) and the sample pad (*t*_SP_) regions is summarized in the table shown in Figure S7b. These regions are highlighted in Figure 2, and the times are averaged across the +BSA and −BSA cases. The times have been scaled to estimate the time taken to flow 4 cm in the nitrocellulose (*t*_NC_*) and the sample pad (*t*_SP_*) regions; this represents the capillary flow time,^42^ allowing comparison to literature values.

Regardless of the buffer used, the time for the liquid front to pass the 1.6 cm central nitrocellulose section of the test was consistently ∼29 s (*t*_NC_). Thus, the interaction time for AuNPCs with the TL was kept constant across the different buffers. However, the time for the liquid to flow through the sample pad, *t*_SP_, varied considerably, and we observed an inverse correlation between *t*_SP_ times and signal at the TL/CL. This indicates that buffers that take longer to pass through the sample pad could be experiencing a loss of AuNPC-CEA-mAb conjugate, thus reducing signal.

Regardless of BSA blocking, the use of PBS (*t*_SP_ = 50.5 s) and Milli-Q (*t*_SP_ = 37 s) does not generate a detectable signal. The inclusion of 0.2% Tween20 to PBS (PBST) (*t*_SP_ = 15 s) increased the flow rate through the sample pad and generated signals, with or without BSA blocking, but also led to nonspecific binding. When using the BioPorto buffer (*t*_SP_ = 14.5 s), the BSA blocking step is required to generate a detectable signal but again generates nonspecific binding. The Innova buffer was included in the panel of buffers tested because of its widescale success as the buffer included in the SARS-CoV-2 rapid test kits. The Innova buffer (*t*_SP_ = 19.5 s) provided the strongest signal at the CL and no nonspecific binding at the TL. This indicates the Innova buffer as the optimal running buffer for our assay. The BSA blocking step was maintained as it is used throughout the literature^35,39^ and may play a more important role when TL binding is in use.

### 3.3 Comparing AuNX Morphologies in CEA LFA Tests

**Figure 3:**
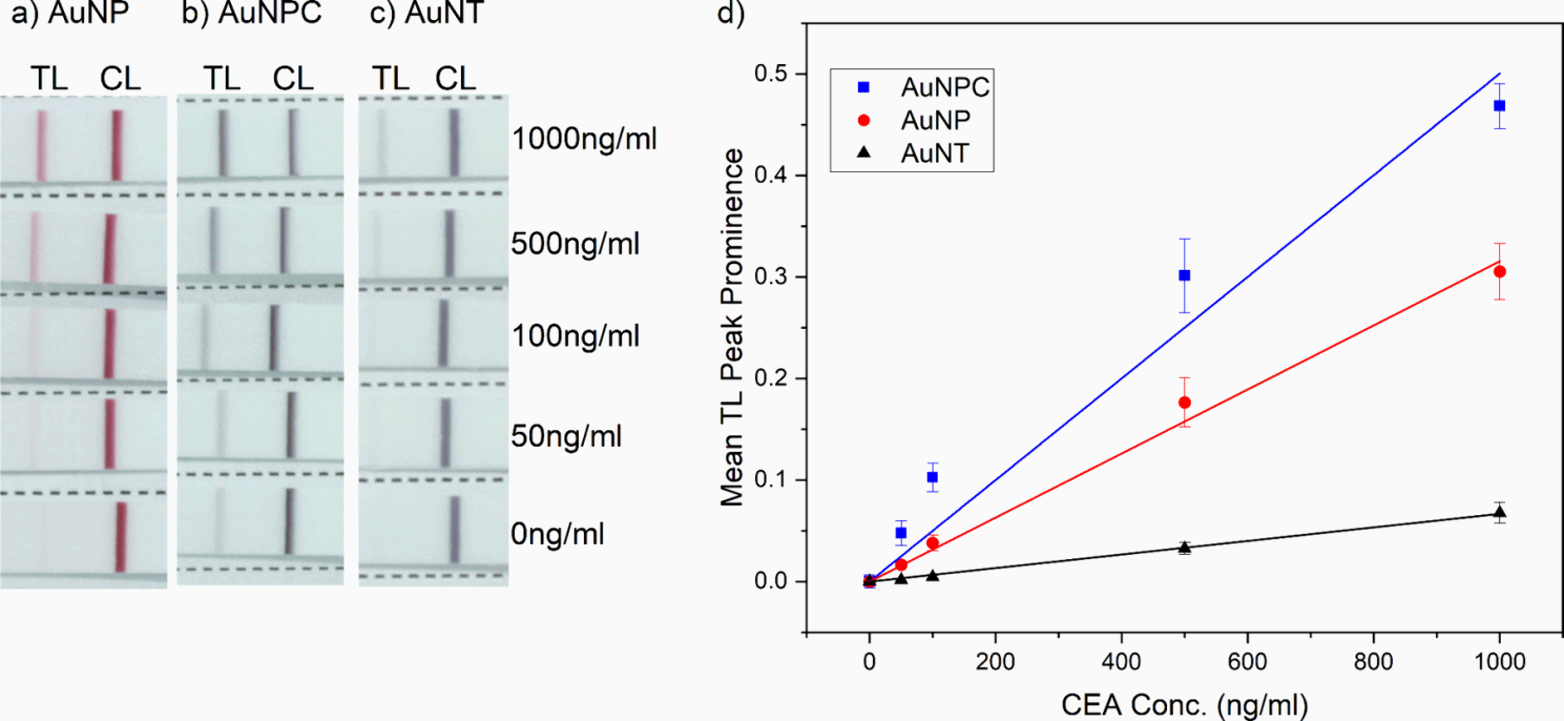
Performance of AuNX in LFA for CEA. Digital photographs of test strips run using a range of CEA concentrations and (a) AuNP, (b) AuNPC, and (c) AuNT. (d) Peak prominence of the TL versus CEA concentration; lines represent linear fits to the data (*n* = 3, error bars represent standard error).

Figure 3a–c shows images of LFA strips detecting CEA, over a range of different concentrations, with different AuNX morphologies. The 40 nm colloidal AuNP generates the classic, deep red signal, whereas AuNPC and AuNT display black/brown and purple signals, respectively. In each case, the total gold concentration was kept fixed by maintaining the same OD_400_ in the conjugation process and in the assay.

Analysis of the peak prominence of the test and control lines (Figure 3d) shows that AuNPC provides the strongest TL signal at all CEA concentrations tested. Hence, AuNPC offers improved CEA sensitivity when compared to commercially available 40 nm spherical AuNPs. The different morphologies show some nonspecific binding, indicating nonoptimized buffer conditions. The level of nonspecific binding in the 0 ng/mL CEA tests was highest for AuNPC, indicating that the high surface area of AuNPC may contribute to unwanted TL binding. In Figure 3d, the peak prominence of the CEA negative control, for each morphology, is subtracted from subsequent peak prominence points. Linear fits to the data provide the gradient and intercepts for calculations of the LOD and LOQ. The data for the comparative assay (collected as described in Section 2.5 and presented in Figure 3) are summarized in Table 1, in which the AuNPC offers the lowest LOD and LOQ while providing the highest gradient. For all AuNX, a corresponding plot of TL/CL peak prominence for each concentration is presented in Figure S8, in which AuNPC demonstrates the greatest gradient followed by AuNP and then AuNT.

**Table 1:** Performance of AuNX in LFA for CEA^a^

morphology	gradient	σ	adjusted *R*^2^	LOD (pg/mL)	LOQ (pg/mL)
AuNPC (comparative)	1.0 × 10^–3^	4.9 × 10^–3^	0.996	15.7k	47.6k
AuNP (comparative)	3.8 × 10^–4^	3.3 × 10^–3^	0.988	28.2k	85.4k
AuNT (comparative)	4.6 × 10^–5^	8.7 × 10^–4^	0.943	62.9k	190.7k
AuNPC (optimized, pre-DAB)	1.9 × 10^–4^	8.4 × 10^–4^	0.995	14.4	43.6
AuNPC (optimized, post-DAB)	1.0 × 10^–3^	9.0 × 10^–3^	0.979	29.1	88.2

a Table showing the gradient, σ (standard deviation of the response), adjusted *R*^2^ value for linear fit, LOD, and LOQ when AuNX were used in LFA for CEA.

AuNPC, AuNP, and AuNT comparative assays showed linear ranges of 50–100, 50–500, and 50–1000 ng/mL respectively. The reproducibility of the assays at different concentrations of CEA is indicated by the error bars in Figure 3d, which represent the standard error of three independent tests, and further by the σ values presented in Table 1. AuNPC, AuNP, and AuNT displayed LOD values of 16, 28, and 63 ng/mL, respectively and LOQ values of 48, 85, and 191 ng/mL, respectively. Hence, for equivalent antibody and gold usage, AuNPC offers a ∼1.8 times reduction in LOD compared to AuNP. As shown in Figure 1e, when diluted to the same OD_400_, AuNP and AuNPC possess the same absorbance at the wavelength of absorbance maximum (OD_peak_). OD_400_ is associated with the total reduced (metallic) gold^43^ and hence was kept fixed across the different morphologies to allow direct comparisons in terms of total gold mass and total assay cost. Atomic absorption spectroscopy (AAS) of triplicate samples showed that AuNT, AuNP, and AuNPC at OD_400_ = 0.5 contained 45.1 ± 1, 43.5 ± 1, and 62.4 ± 1 μg/mL gold, respectively. At fixed OD_400_, the nanoparticle number varies between the AuNX. Nanoparticle tracking analysis (NTA) indicated that 8.4 × 10^8^ AuNPCs were used in each comparative LFA compared to 2.7 × 10^9^ AuNPs. Hence, AuNPCs provided improved signal in comparison to AuNPs when used at 3.2× less particle number. Fixing the particle number would potentially show a greater improvement afforded by the AuNPCs compared to AuNPs. The poorer performance of the AuNTs is most likely because, for the same OD_400_, AuNTs have the lowest integrated peak absorbance, as shown in Figure 1e.

### 3.4 Enhancing LFA Sensitivity Using the DAB Substrate

Previously, we have shown that AuNTs and AuNPCs exhibit a nanoenzymatic behavior.^34^ In particular, our 2D AuNX constructs can be used to oxidize DAB, in the presence of H_2_O_2_, to produce a polymer that has the form of a brown/black powder. As AuNPC offers the greatest LFA sensitivity, the AuNPC assay was selected for further development. The bio-mAb concentration used was increased to 100 μg/mL, and a buffer “wash” step that consisted of the addition of 50 μL of the BioPorto buffer was included following the addition of the sample solution, with the aim of pulling any residual AuNPC-antibody conjugates retained in the sample pad or NC membrane to the wicking pad. The DAB substrate enhancement step consisted of adding 100 μL of a DAB/H_2_O_2_ reaction solution.

**Figure 4:**
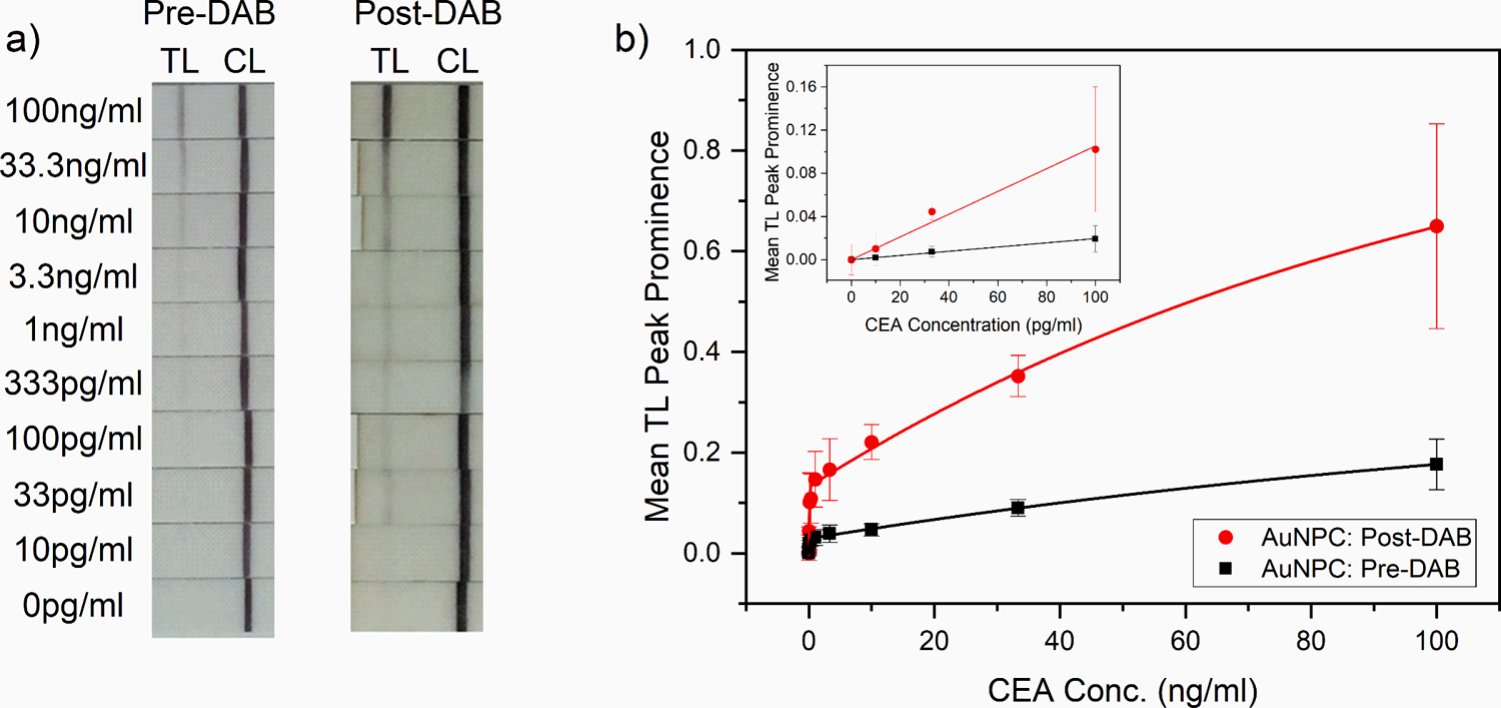
Performance of AuNPC in LFA for CEA with DAB amplification**.** (a) Digital photographs of test strips run using a range of CEA concentrations and AuNPC, pre- and post-DAB. (b) Peak prominence of the TL versus CEA concentration. The inset shows the initial increase in peak prominence for low concentrations of CEA; lines are linear fits to the data (*n* = 3, error bars represent standard error).

Figure 4a shows images of AuNPC-based LFA strips before and after signal amplification using DAB. A clear enhancement of both the TL and CL signal is observed. Figure 4b shows the peak prominence analysis, with the inset showing a close-up view of the 0–100 pg/mL data. In Figure 4b, the peak prominence of the CEA negative control was subtracted from subsequent peak prominence points. The 0–100 pg/mL range data were used to calculate the gradient, LOD, and LOQ, with data for the optimized assay (collected as described in Section 2.6 and presented in Figure 4) summarized in Table 1. The assay appears to consist of two regimes, similar to the findings of Gao et al.^35^

The pre- and post-DAB optimized AuNPC assays both exhibited linear ranges of 10–100 pg/mL. The reproducibility of the assays at the different concentrations of CEA is indicated by the error bars in Figure 4b, which represent the standard error of three independent tests, and further by the σ values presented in Table 1. Pre-DAB, AuNPCs displayed an LOD of 14.4 pg/mL and an LOQ of 43.6 pg/mL. Post-DAB, AuNPCs displayed an LOD of 29.1 pg/mL and an LOQ of 88.2 pg/mL. The higher LOD post-DAB is due to the increase in the standard deviation associated with the data, arising from the increased variance measured in the post-DAB signals. However, from a practical standpoint, the addition of DAB provides a ∼ 4–5× enhancement for signals for 3.3 and 10 ng, in the clinically relevant range, aiding visual assessment.

## 4 Conclusions

Our studies show that the high surface area AuNPCs exhibited a ∼2× improvement in LFA LOD in comparison to commercially available 40 nm AuNPs for the same antibody loading and total gold content, whereas the number of AuNPCs in each test was ∼3.2× less. Further, the high surface area and edge facets of the AuNPCs (90 nm in diameter) provide nanoenzymatic properties that potentially offer the observed improvement in sensitivity over the conventionally used spherical AuNPs.

Interestingly, the optimized AuNPC lateral flow device without DAB enhancement gave a CEA LOD of 14.4 pg/mL. This represents a 24-fold lower LOD compared to other AuNP-based LFAs^26^ and a 14-fold reduction in LOD compared to commercially available ELISA kits for CEA.^16^ Although DAB amplification increased the peak prominence and the gradient of the data (and hence sensitivity, see Table 1) the LOD, as defined by eq 1, resulted in a higher LOD due to the increase in standard deviation between samples. The improved LOD of our system is coupled with a simple method that is applicable in a range of settings, uses nonhazardous reagents/materials (in the absence of DAB), requires minimal equipment, and can be completed in ∼15 min. Although the focus of this work was demonstrating the enhanced performance of novel nanoparticles, areas for future work include the optimization of buffer conditions to minimize nonspecific binding, the use of predried conjugate pads and test/control lines to allow easier storage/use, and the optimization of the test for analysis of CEA-spiked human serum.

## Supporting Information Available

AuNPC TEM and sizing data (Figure S1); colloidal stability of AuNPC and AuNT (Figure S2); representative intensity profiles obtained from AuNPC LFA (Figure S3); absorbance spectra of each stage of the conjugation of AuNX to anti-CEA mAb (Figure S4); optimizing AuNP-antibody conjugate concentration (Figure S5); optimization of biotinylated antibody concentration (Figure S6); selection of optimal running buffer and influence of BSA blocking step (Figure S7); and plot of TL/CL for each CEA concentration in the comparative assay (Figure S8). Data used in the figures of this paper are openly available from the University of Leeds data repository 10.5518/1173 (PDF)

## ABBREVIATIONS

CRC: colorectal cancer
CEA: carcinoembryonic antigen
ELISA: enzyme-linked immunosorbent assay
AuNP: gold nanoparticle
LOD: limit of detection
LFA: lateral flow assay
TL: test line
CL: control line
AuNS: gold nanosheet
AuNT: gold nanotape
AuNPC: gold nanopinecone
TMB: tetramethylbenzidine
DAB: diaminobenzidine
MO: methyl orange
UV–vis-NIR: ultraviolet–visible-near-infrared spectroscopy
TEM: transmission electron microscopy
SEM: scanning electron microscopy
AAS: atomic absorption spectroscopy
mAb: monoclonal antibody
OD: optical density
LOQ: limit of quantification.
