# Supplementary material for: Gold Nanotapes and Nanopinecones in a Quantitative Lateral Flow Assay for the Cancer Biomarker Carcinoembryonic Antigen
Source: ACS Appl Nano Mater. 2023 Sep 19;6(19):17769–77. doi: 10.1021/acsanm.3c03053 (PMC10580237; doi:10.1021/acsanm.3c03053)
Supplement: Supplementary file 1 — an3c03053_si_001.pdf [file an3c03053_si_001.pdf]

Supporting Information

Gold Nanotapes and Nanopinecones in a Quantitative Lateral Flow Assay for the Cancer Biomarker Carcinoembryonic Antigen

Joseph Fox[1], Damien V. B. Batchelor[1], Holly Roberts[1], Samuel C.T. Moorcroft[1], Elizabeth M.A. Valleley[2], Patricia Louise Coletta[2] and Stephen D. Evans[1].*

[1]Molecular and Nanoscale Physics Group, School of Physics and Astronomy, University of Leeds, LS2 9JT, United Kingdom

[2]Leeds Institute of Medical Research, Wellcome Trust Brenner Building, St James's University Hospital, Leeds, LS9 7TF, United Kingdom

*Corresponding author E-mail: s.d.evans@leeds.ac.uk

1 Supporting Information

1.1 AuNPC TEM and Sizing Data

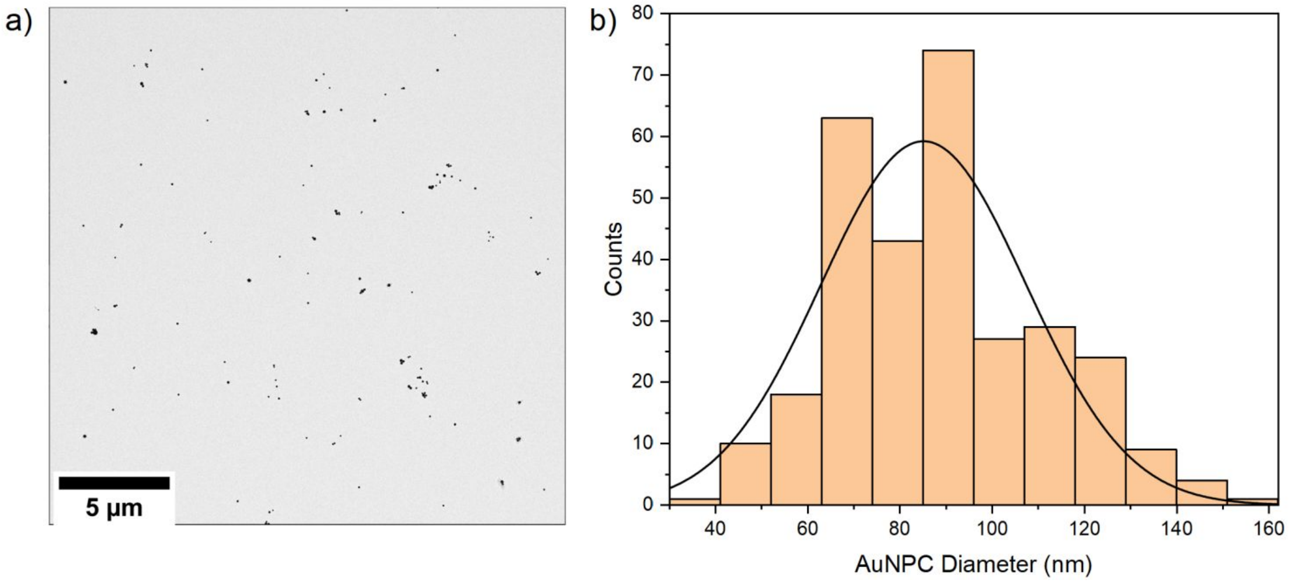

Figure S1 AuNPC TEM and Sizing Data. a) Representative widefield TEM of AuNPC sample. The image shown is representative of the image magnification and scale used for the ImageJ sizing analysis. b) Sizing data obtained by ImageJ analysis of multiple widefield TEM images, N = 303, Gaussian fit applied.

1.2 Colloidal Stability of AuNPC and AuNT

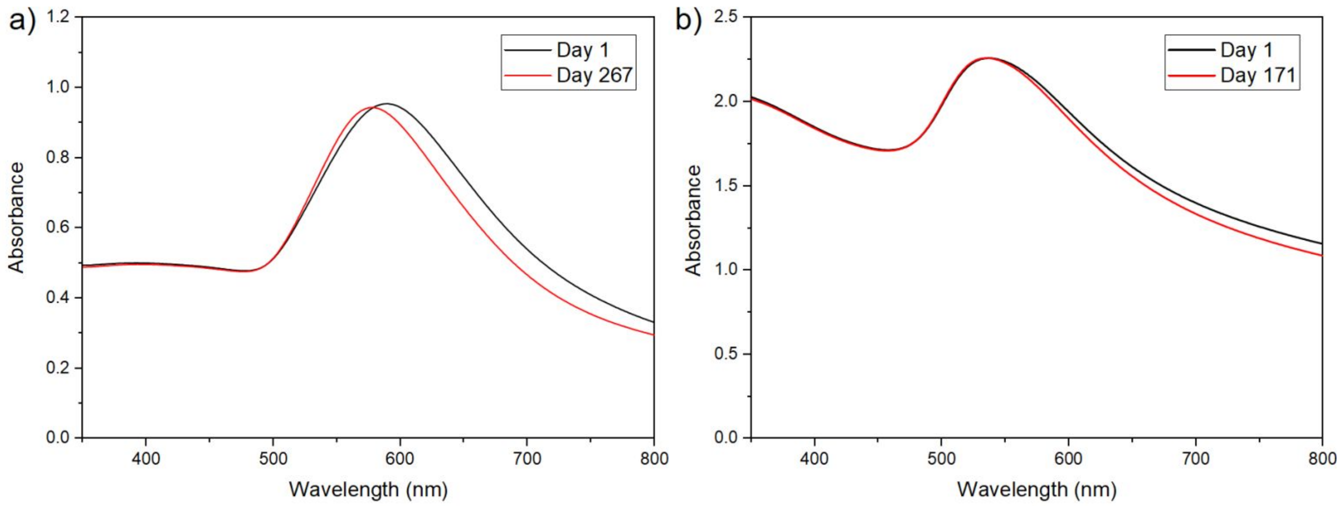

Figure S2 Colloidal Stability of AuNPC and AuNT. a) UV-Vis absorbance spectra of AuNPC stored at ambient conditions, spectra obtained on day 1 and 267. b) UV-Vis absorbance spectra of AuNT stored at stored at ambient conditions, spectra obtained on day 1 and 171.

1.3 Strip Signal Analysis

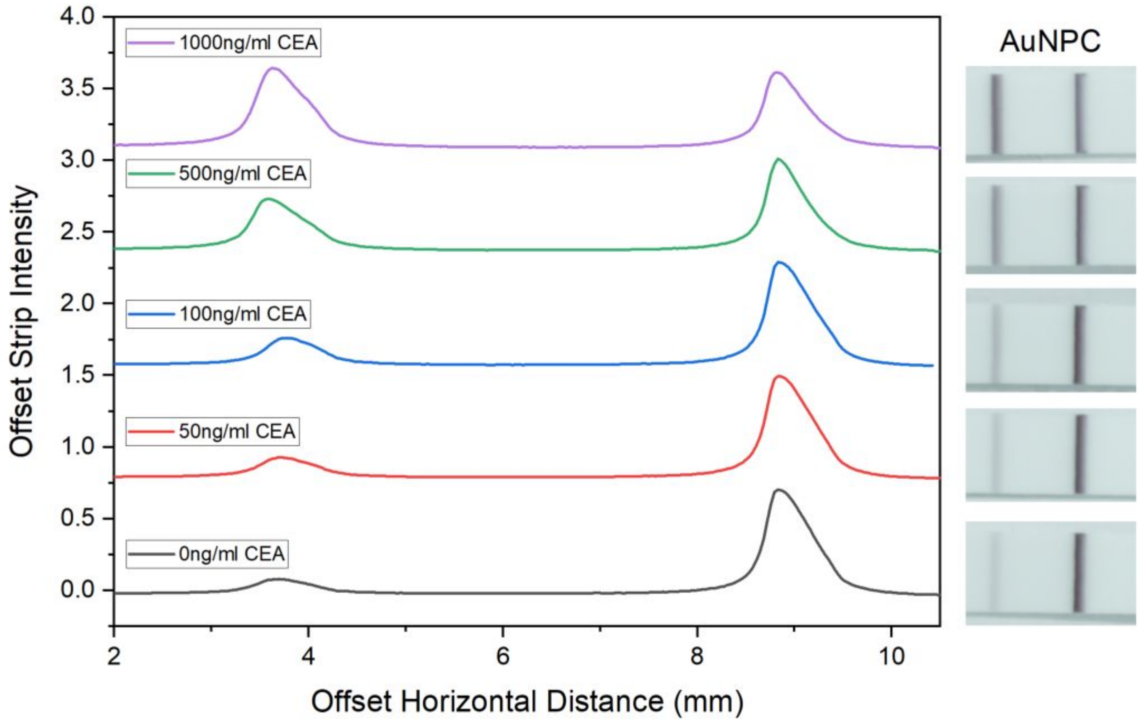

Figure S3 Representative intensity profiles obtained from AuNPC LFA. Analysis was performed using a custom MATLAB script to analyze the pixel intensity of a horizontal profile across each LFA strip. Color images were taken using a Canon EOS 550D DSLR camera, fixed in place in a light box to ensure consistent lighting of each image. Camera acquisition settings were constant across all images and colour images converted to grayscale by averaging intensity over RGB channels. LFA strips were placed inside a calibration square containing white and black boxes, which were used to determine the absolute brightness of the image. This was subsequently used to normalize image intensity of each image and strip before profile

analysis, such that an intensity of 0 corresponds to absolute white (white calibration box) and 1 corresponds to absolute black (black calibration box). Peak prominence for both control and test lines was then found to quantify intensity.

1.4 Conjugation of AuNX to Anti-CEA Monoclonal Antibodies

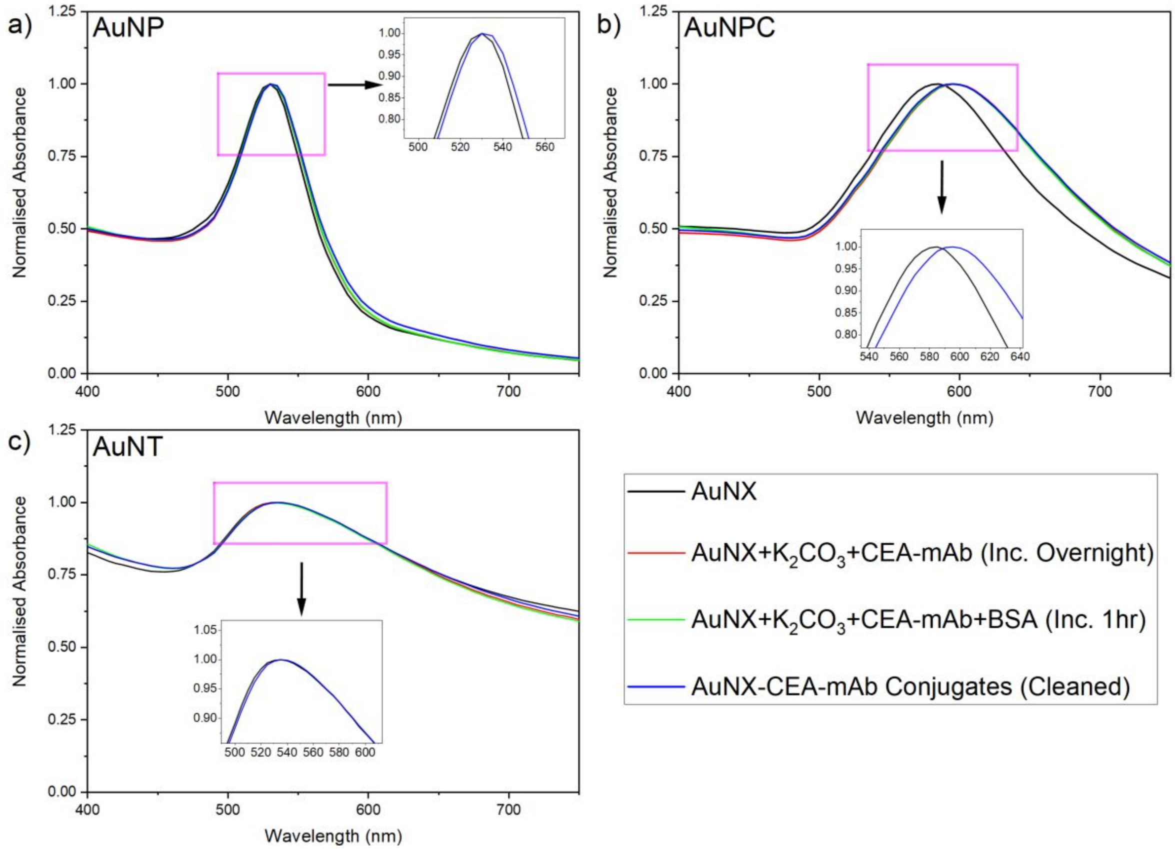

Figure S4 Absorbance Spectra of Each Stage of the Conjugation of AuNX to Anti-CEA mAb.

Plots directly compare absorbance spectra of unconjugated AuNX solution (black line), AuNX after overnight incubation with K_2CO_3 and CEA-mAb (red line), AuNX+K_2CO_3+CEA-mAb

after 1 hour incubation with BSA blocking solution (green line) and AuNX+K_2CO_3+CEA-mAb+BSA after cleaning by centrifugation to produce cleaned AuNX-CEA-mAb conjugates (blue line). Spectra shown with peak absorbance normalized to 1. Insets show zoomed view of the absorbance peak of unconjugated AuNX and the final AuNX-CEA-mAb conjugates post cleaning, in which the morphologies are a) AuNP, b) AuNPC and c) AuNT.

1.5 Optimising AuNP-Antibody Conjugate Concentration used in LFA

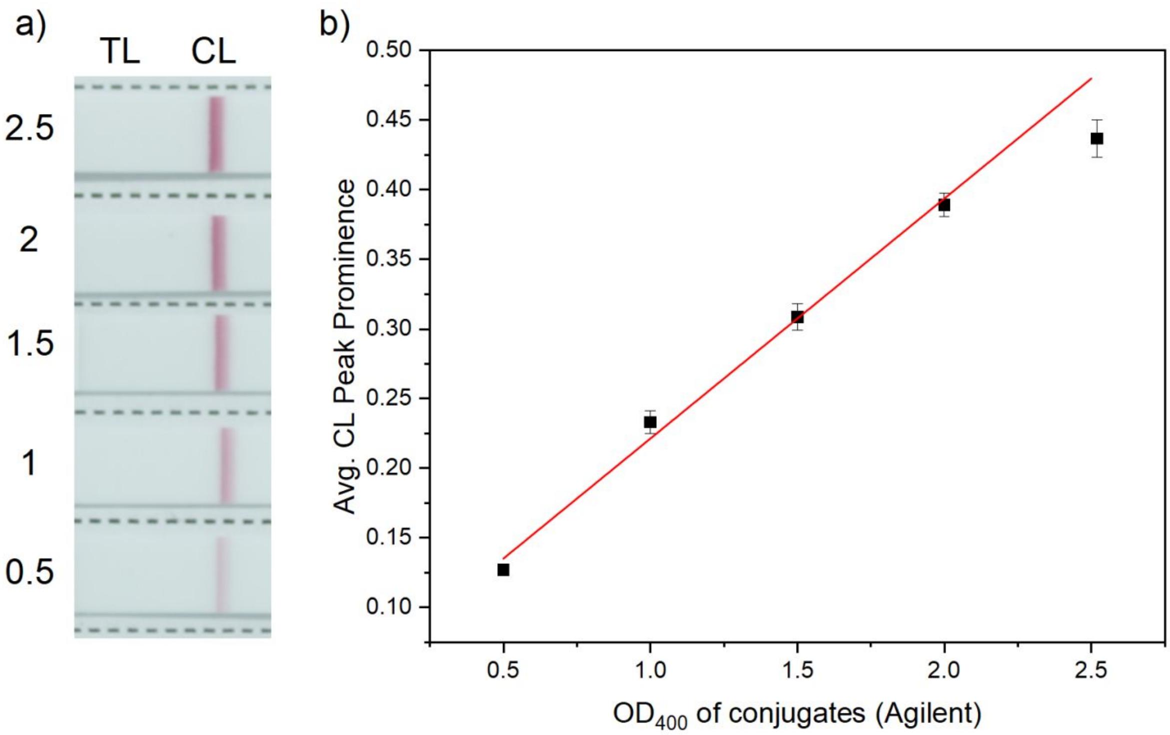

Figure S5 Optimizing AuNP-antibody conjugate concentration. Optimization conducted using AuNP conjugated to rabbit IgG polyclonal antibody (PP64-pAb). LFA undertaken by dipping the bottom of the test strip in a 96-well plate containing 10 µL AuNP-PP64 conjugates diluted to provide a range of different OD_{400} (Agilent) and 100 µL BioPorto buffer. No TL binding occurs for any concentration tested, as the secondary biotinylated antibody needed to permit binding at the TL was not used in this test. a) Digital photographs of test strips run using different concentrations of AuNP-antibody conjugate, run with blank BioPorto buffer. b) Corresponding peak prominence analysis of the CL. Linear fit applied, omitting the OD_{400} = 2.5 point (n=3, error bars represent standard error (SE)).

1.6 Optimizing Biotinylated Antibody Concentration

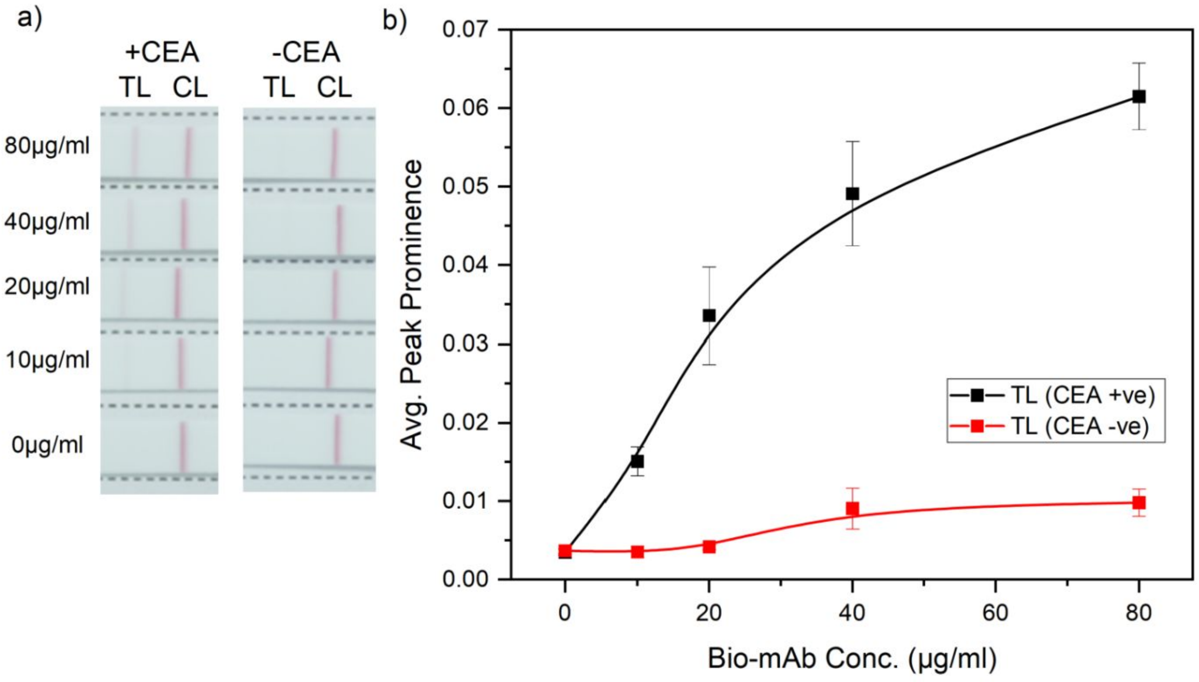

Figure S6 Optimization of biotinylated antibody concentration. Optimization conducted using AuNP conjugated to anti-CEA-mAb. LFA undertaken by dipping the bottom of the test strip in a 96-well plate containing 5 µL bio-mAb at a range of concentrations, 10 µL AuNP-CEA-mAb conjugates (OD_{400}=1.5, Agilent) and 100 µL CEA at 1000 ng/mL in BioPorto buffer. Blank BioPorto buffer was used as a CEA negative control. a) Digital photographs of test strips run using different concentrations of bio-mAb. Tests carried out with buffer spiked with 1000

ng/mL CEA (+CEA) and 0 ng/mL CEA (-CEA). b) Corresponding peak prominence analysis of the TL and CL for the +CEA and -CEA samples when a range of bio-mAb concentrations used in the assay (n=3, error bars represent SE). B-splines applied to TL data.

1.7 Running Buffer Selection and Importance of BSA Blocking on LFA Performance

a)

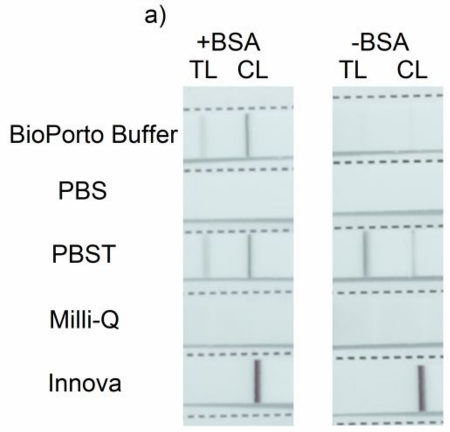

b)

t_{NC}(s)	t_{NC}^{*}(s)	t_{SP}(s)	t_{SP}^{*}(s)
29 ± 0	72.5 ± 0	14.5 ± 0.5	21.5 ± 1
27 ± 0	67.5 ± 0	50.5 ± 6	74.7 ± 8
29 ± 1	72.5 ± 3	15 ± 2	22.2 ± 3
26.5 ± 1	66.3 ± 1	37 ± 4	54.8 ± 6
31.5 ± 2	78.8 ± 4	19.5 ± 1	28.9 ± 1

Figure S7 Selection of optimal running buffer and influence of BSA blocking step. Optimization conducted using AuNPC conjugated to PP64-pAb, with and without the inclusion of the BSA blocking step during the conjugation. LFA undertaken by dipping the bottom of the test strip in a 96-well plate containing 10 μL AuNPC-PP64 conjugates (OD_{400} =3.6, Agilent) and 100 μL running buffer. We only expect CL binding, as no secondary antibody has been

used to enable TL binding, hence any TL signal is non-specific. a) Digital photographs of test strips run using different running buffers. And using AuNPC-PP64 conjugates with (+BSA) and without (-BSA) a BSA blocking step during the conjugation procedure. b) Time taken for material to flow through the nitrocellulose membrane (t_{NC}) and sample pad (t_{SP}) regions of the test strip. Table also includes time taken to flow 4 cm in the nitrocellulose membrane (t_{NC}^{*}) and sample pad (t_{SP}^{*})

1.8 Plot of TL/CL

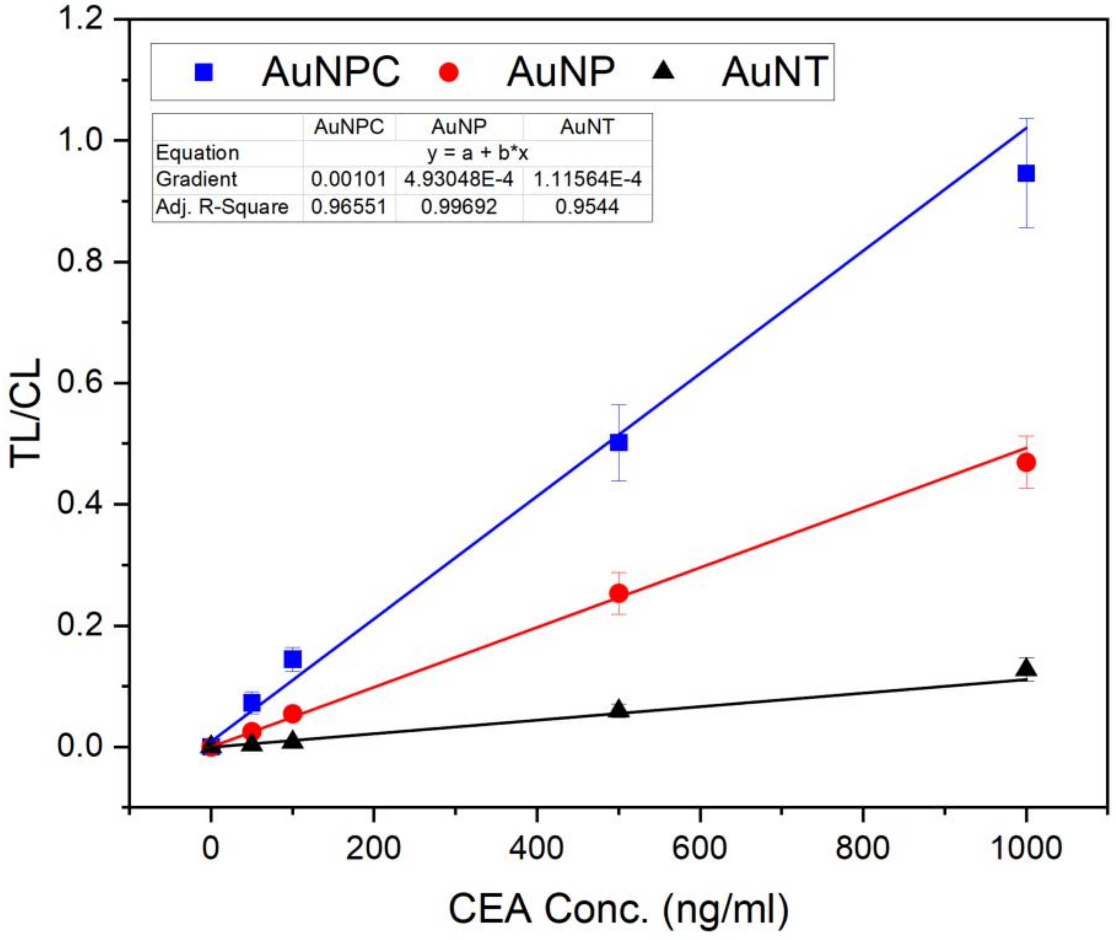

Figure S8 Plot of TL/CL for each CEA Concentration in the Comparative Assay. Ratio of TL and CL peak prominence plotted for each concentration of CEA for each AuNX. Lines represent linear fits to the data (n=3, error bars represent standard error).
